# From Antenna to Antenna: Lateral Shift of Olfactory Memory Recall by Honeybees

**DOI:** 10.1371/journal.pone.0002340

**Published:** 2008-06-04

**Authors:** Lesley J. Rogers, Giorgio Vallortigara

**Affiliations:** 1 Centre for Neuroscience and Animal Behaviour, University of New England, Armidale, Australia; 2 Centre for Mind/Brain Sciences, University of Trento, Rovereto, Italy; University of Cambridge, United Kingdom

## Abstract

Honeybees, *Apis mellifera*, readily learn to associate odours with sugar rewards and we show here that recall of the olfactory memory, as demonstrated by the bee extending its proboscis when presented with the trained odour, involves first the right and then the left antenna. At 1–2 hour after training using both antennae, recall is possible mainly when the bee uses its right antenna but by 6 hours after training a lateral shift has occurred and the memory can now be recalled mainly when the left antenna is in use. Long-term memory one day after training is also accessed mainly via the left antenna. This time-dependent shift from right to left antenna is also seen as side biases in responding to odour presented to the bee's left or right side. Hence, not only are the cellular events of memory formation similar in bees and vertebrate species but also the lateralized networks involved may be similar. These findings therefore seem to call for remarkable parallel evolution and suggest that the proper functioning of memory formation in a bilateral animal, either vertebrate or invertebrate, requires lateralization of processing.

## Introduction

Bees form olfactory memories of the scents of flowers from which they have obtained nectar and this can be demonstrated easily using the proboscis extension reflex (PER). In just three trials a bee will learn to associate an odour, such as lemon, vanilla or geraniol, with a sugar reward and will extend its proboscis when the odour alone is presented [1], [2]. By coating either the left or right antenna with latex and thus rendering one antenna incapable of detecting odour, Letzkus et al. [3] were able to show that honeybees learn well when they use the right antenna but poorly if they use the left antenna.

This finding of lateralization in the bee numbers amongst the handful of studies showing that invertebrate species may be lateralized, similar to the widespread lateralization of the nervous system in vertebrates [4], [5], [6]. Other examples of lateralization in invertebrate species include a side bias seen in spitting spiders, *Scytodes globula*, to probe potential prey with the front legs on the left side [7] and for this and many other species of spiders and ants to sustain injury to legs on the left side [8]. An asymmetrical neural structure in the fruitfly brain is coincident with the ability to form long-term memories [9], which indicates an advantage of lateralization as also found in the domestic chick [10].

It is beginning to look as if lateralization of the nervous system is a feature of simpler brains as well as more complex ones. Hence, careful observation of the behaviour of insects may reveal more examples of lateralization even in their natural behaviour in the wild. In fact, Kells and Goulson [11] have reported that bumble bees, *Bombus spp.,* show preferred directions of circling as they visit florets arranged in circles around a vertical inflorescence. In three out of four species examined the majority of bumble bees circled in the same direction. Since two species circled anticlockwise and one clockwise, it is likely that the asymmetry was a function of the bees and not the structure of the florets. In fact, the circling might well have something to do with lateralization of the antennal responsiveness to odours.

We were interested in finding out whether the lateralization of olfactory learning demonstrated in honeybees could be seen in recall of memory at various times after the bees had been trained using both antenna and also to see whether such lateralities would be manifested as side biases to odours in bees tested with both antennae functional, and so in a more natural condition than the paradigm requiring one antenna to be coated with latex.

## Results and Discussion

In the first experiment we looked at recall when bees used only their left or right antenna after training with both antennae. Bees were trained using lemon plus sucrose solution as the positive stimulus and vanilla plus saturated saline as the negative stimulus. Within 5–10 mins after training one antenna was coated (left or right antenna chosen at random) and retention was tested 1 hour later by presenting lemon or vanilla solutions in distilled water. The responses were scored as follows: A, extension of the proboscis to lemon and no extension to vanilla; B, extension of the proboscis to both lemon and vanilla; C, extension of the proboscis to vanilla but not lemon; D, no extension of the proboscis to lemon or to vanilla. By obtaining 10 scores per bee we controlled for consistency of responses for each bee and could use smaller sample sizes than used previously [3].

Immediately after the 10 retention trials were completed the coating was removed from the antenna and the other antenna was coated. One hour after this operation the retention test was repeated.

A total of 12 bees were tested for recall at 1–2 hour after training, 7 tested with the right antenna coated first and 5 tested with the left antenna coated first. A further 14 bees were tested for recall at 23–24 hours after training, 6 with the right antenna tested first and 8 with the left first. In the latter tests, one of the bee's antennae was coated at 22 hours, recall was tested and then the cover was removed immediately and the other antenna coated, followed by recall testing at 24 hours.

**Figure 1:**
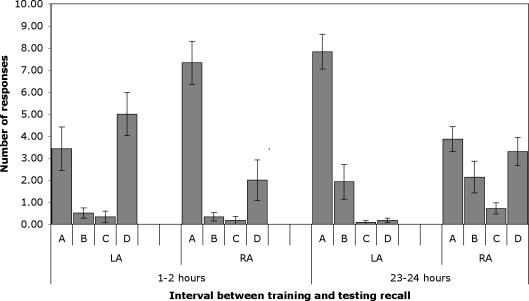
Experiment 1: Recall of memory at 1–2 and 23–24 hours after training. All bees were trained using both antennae and tested for recall using either the left (LA) or right antenna (RA), the other being covered with latex. The mean number (±standard error) of type A responses (proboscis extension response, PER, to lemon odour and not vanilla odour), B (PER to both odours), C(PER to vanilla and not lemon) and D (no PER) are plotted. The A scores were analysed by GLM using the factors Antenna (left versus right antenna in use) and Order (left antenna tested first or right antenna tested first). At 1–2 hours after training, there was no significant main effect of Order (F(1,20) = 2.896, p = 0.104) and no significant interaction between Order and Antenna (F(1,20) = 0.478, p = 0.479). The main effect of Antenna was significant (F(1,20) = 7.358, p = 0.013). A responses were significantly higher when the right antenna was in use than when the left antenna was in use. At 23–24 hours after training, there was no significant main effect of Order (F(1,22) = 0.124, p = 0.728) and no significant interaction between Order and Antenna (F(1,22) = 1.597, p = 0.220). The main effect of Antenna was significant (F(1,22) = 14.064, p = 0.001). A responses were significantly higher when the left antenna was in use than when the right was in use.

The scores analysed were total number of A responses per bee (maximum possible A scores per bee was 10). At 1–2 hours after training, A responses were significantly higher when the right antenna was in use than when the left antenna was in use (results of data analysis in footnote to Figure 1). At 23–24 hours after training, A responses we significantly higher when the left antenna was in use than when the right was in use (Figure 1). Hence relative ease of access to memory shifts from antenna to antenna.

D scores made up by far the majority of non-A responses, both B and C responses being rare, apart from a slight but significant increase in B responses at 23–24 hours in both the LA and RA conditions (significant only for RA; 2-tailed t-test, p = 0.04). This increase represents increased errors at the 23–24 hour interval.

Letzkus et al. [3] restricted stimulation to only one antenna during both training and recall testing 24 hours later and found better performance with the right antenna. Better performance with the right antenna in this case is likely to be due to the restricted use of one antenna only during training. Learning with both antennae, as in our test, allows competition between inputs to both sides of the brain and, as shown previously [12], increases processing capacity. Both these aspects may be needed for time-dependent shifts in lateral biases to take place.

**Figure 2:**
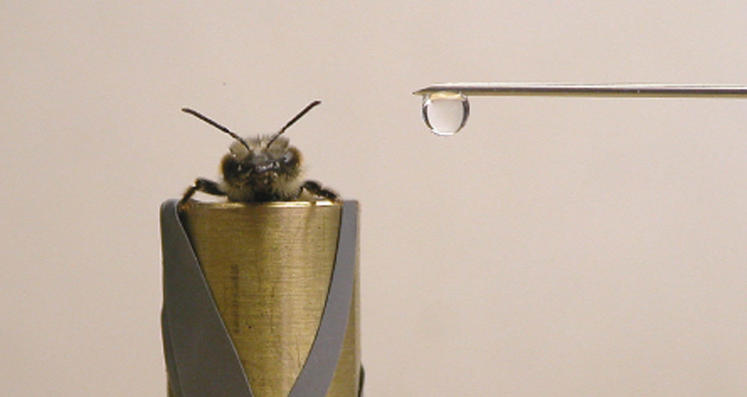
A photograph of lateral presentation of the odour as in experiment 2.

In the second experiment we looked at recall at several intervals after training both antennae and by testing using lateral presentation and no coating of the antennae. The odour was presented to the left or right side of the bee (Figure 2). The exhaust fan would have ensured that the antenna closer to the droplet would have received a higher concentration of odour reaching the antenna further from it. Moreover the bee moved only the closer antenna as close as possible to the droplet. A total of 29 bees were trained with lemon sugar and vanilla. A total of 18 bees were tested for recall at either 1, 3 or 6 hours after training and a further 11 bees were tested 23 hours after training.

In addition to calculating the total number of A, B, C and D scores as in Experiment 1, a Laterality Index (LI) was calculated for each bee, using (L−R)/(L+R), where L represents the number of proboscis extensions on presentation of lemon water on the left side and R the number of PER on presentations of lemon water on the right side.

**Figure 3:**
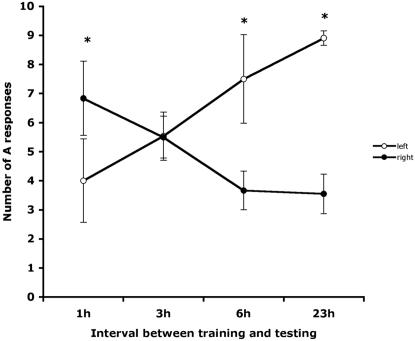
The mean number of A responses, plotted with standard error bars, in recall tested at various intervals after training is shown for presentations of the odours on the bee's left side (open circles) or right side (closed circles).

The data for number of A responses (extension to lemon and not to vanilla) were analysed by GLM with side as a repeated measure and time as a factor. There was a significant main effect of side (F_1,25_ = 10.452, p = 0.003) and a significant interaction between side and time of testing (F_3,25_ = 13.709, p = 0.0001); Figure 3. Post hoc paired t-tests between the left and right sides showed that there were significantly more A responses on the right than on the left side at 1 hour after training (1-tailed, paired t-test, p = 0.045), replicating the results of experiment 1 using the different method of testing. No significant left/right difference in A scores occurred at 3 hours after training (p = 0.768) and this was due to the number of A responses being high on both sides of presentation. At both 6 and 23 hours after training the left/right difference was significant (p = 0.016 and p = 0.0001, respectively, 2-tailed paired t-tests) and at these times the A responses were higher on the left side than on the right side. The non-A responses were almost entirely D responses (i.e. no PER for either lemon or vanilla).

**Figure 4:**
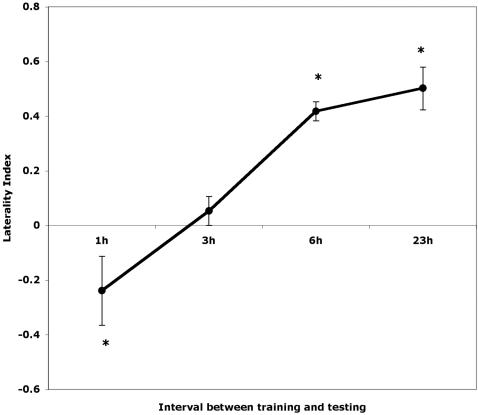
Experiment 2: The laterality Index is plotted as means+standard errors at different intervals after training. Asterisks indicate significant divergence from no bias. Note the shift from a right side bias at 1 hour to a left side bias at 6 and 23 hours. The LI scores were analysed by ANOVA using time of testing as a factor. A significant effect of time of testing was found (F_3,25_ = 16.769, p = 0.0001). Post hoc LSD tests showed that the LI scores at 1 hour after training differed significantly from those at 3 hours (p = 0.046), 6 hours (p = 0.0001) and 23 hours (p = 0.0001). The LI at 1 hour was biased to the right side, whereas at 6 and 23 hours the bias was to the left side and the same at both times (p = 0.473). At 3 hours after training the LI had no significant bias as responding was equal on both sides and differed significantly from LI at 6 hours (p = 0.007) and 23 hours (p = 0.0001).

LI scores are presented in Fig. 4, showing a right antenna bias at 1 hour after training, no bias at 3 hours after training and a left bias at 6 and 23 hours after training (statistical results in footnote of Figure 4).

One hour after training with both antennae in use, the bees showed excellent recall of the task when they were tested using the right antenna but poor or no recall when they were using the left antenna. Hence, not only is learning better when the right antenna only is in use than when the left antenna only is in use [3] but also recall 1–2 hours after training is better when the bee uses its right antenna than when it uses its left antenna. This role of the right antenna in memory recall is, however, transient: longer-term recall relies on use of the left but not the right antenna. Such a shift of recall from one to the other side of the brain has been noted previously in birds [13], [14], [15]. Hence, not only are the cellular events of memory formation similar in bees and vertebrate species [16] but also the lateralized processes involved may be similar.

It appears that the time-dependent recall of odour memories is lateralized with the transition from shorter-term recall via the right antenna to long-term memory recall via the left antenna taking place at about 3 hours after training. Although similar neural structures might be involved in memory formation on the left and right sides, it seems that antennal inputs may access different neural circuits on the left and right sides. In fruitflies, for example, the asymmetrical structure that enhances long-term memory recall is on the right side of the brain [9].

Associative olfactory learning in bees involves both unilateral and bilateral neural processes [17] and the mushroom bodies on each side of the brain are involved in memory formation following unilateral input [18]. Moreover, odours evoke symmetrical patterns of glomerular activity [19]. Hence it is possible that learning via the right antenna is sufficient to trigger shorter-term encoding on the right side and longer-term encoding on the left side. Alternatively, the memory encoding is the same on both sides of the brain but only the right antenna has access for shorter-term recall and only the left antenna has access for longer-term recall. Using previous terminology for the stages of memory recall, our shorter-term recall via the right antenna is referred to as mid-term memory, whereas the 23-hour recall is definitely long-term memory [16].

Perhaps the shift from one antenna to the other allows use of the right antenna to learn about new odours without interference from odour memories in long-term stores. It is known that bees visit different flowers at different times of the day, as nectar becomes available [20], and this would require the formation of different odour associations during the course of the day, a process that might be aided if recall of earlier odour memories is avoided on the learning side of the brain.

The side biases found without any coating of the antenna appear to depend on relative differences in the concentration of odour reaching each antenna. Although bees show lateralized visual responsiveness [21], we were able to exclude visual lateralization as a factor contributing to our results by a third experiment, in which droplets on the needles of syringes were presented simultaneously on both sides of the bee, one droplet being distilled water only and one containing the odour. The results were the same as obtained in experiment 2: 7 bees tested at 1 hour after training showed a right side bias (mean LI index±sem, −0.65±0.23; significant departure from chance level was estimated by one-sample two-tailed t test: t(6) = 2.826 p<0.05) and 6 bees tested 23 hours after training showed a left side bias (LI, +0.50±0.18; one-sample two-tailed t test: t(5) = 2.778 p<0.05).

Since the lateral responsiveness to odours is shown without any coating of the antenna, it is likely that it is manifested as side biases in natural foraging behaviour. Indeed, it may be the explanation for the circling seen in bumblebees as they visit florets [11]. It would be interesting to see whether such circling occurs in *Apis mellifera* and possibly the direction would be clockwise, using the right antenna, on first exposure to a particular flower's odour and anticlockwise, using the left antenna, after a delay period. Lateralized responsiveness to odours may also be important in the known time-dependent changes in foraging at different flowers [20] and the accuracy of rhythms in foraging [22].

In the very least, our results show that future research involving odour conditioning of single antennae should take side differences into account and not assume that they can ignore left-right differences if groups are simply balanced for left and right antennal training [12], [19]. More importantly, our findings suggest that functioning of the nervous system of a bilateral animal, whether vertebrate or invertebrate, involves lateralization of processing. Lateralization appears to be a necessary, or very advantageous, feature of any brain with paired sensory organs.

## Materials and Methods

Bees were captured when foraging at around 9.00h, cooled in 750 ml containers until immobilised and then secured in holders, using the method of Bitterman et al. [1] but with the addition of a small strip of paper to cover the bee's back so that it would not be damaged by the adhesive tape and could be released after testing. After 1 hour each bee was placed in front of an exhaust fan and trained using lemon plus 1M sucrose solution as the positive stimulus and vanilla plus saturated saline as the negative stimulus (10ul of lemon or vanilla essence was dissolved in 3 ml of the solutions). Three trials spaced 6 mins apart were given. On the first trial a droplet of the lemon sugar solution at the end of a 23 gauge needle was held over the bee's antennae at 1 cm from the antennae and, after 5 seconds the antennae were touched, which led to the proboscis extension response (PER). The bee was then allowed to ingest the drop of lemon sugar solution. Immediately after this, the procedure was repeated with the vanilla saline solution, which did not trigger PER but avoidance by moving the antennae away from the droplet. Trial 2 commenced 6 min later and usually PER occurred without the need to touch the antennae, followed by trial 3 after another 6 min later. All bees were trained in the same way and subsequently assigned randomly to groups for occlusion of one antenna and retention testing.

In the experiment testing recall 1–2 hours after training, within 5–10 mins after training one antenna was coated (left or right antenna chosen at random) with Silagum-Mono (Chemisch-Pharmazeutische Fabik Cmbtt, Germany). Retention was tested 1 hour later by presenting lemon or vanilla solutions in distilled water and holding the droplet 1 cm from the antennae while moving it slightly but being sure not to touch the antennae. Vanilla water was presented for 5 secs followed by lemon water for the same time and this was repeated 10 times at intervals of approximately 60 sec. Previous tests had shown that no habituation of the PER response occurs over 20 such trials. The responses were scored as follows: A, extension of the proboscis to lemon and no extension to saline; B, extension of the proboscis to both lemon and vanilla; C, extension of the proboscis to vanilla but not lemon; D, no extension of the proboscis to lemon or to vanilla. By obtaining 10 scores per bee we controlled for consistency of responses for each bee and could use smaller sample sizes than used previously [3].

Immediately after the 10 retention trials were completed the Silagum coating was removed from the antenna by pulling an edge of it very gently using fine forceps and viewing under a binocular microscope. Next the other antenna was coated with Silagum. If the procedure caused any damage to the antenna that bee was discarded but this was very rare. One hour after this operation the retention test was repeated.

When recall was tested at 23–24 hours after training, the coating of first one antenna and then the other was carried out at 22 and 23 hours respectively (i.e. 1 hour before each recall test).

In the second experiment we looked at recall at several intervals after training both antennae and by testing using lateral presentation and no coating of the antennae. The bee was again placed in front of an exhaust fan and odour was presented to the left or right side of the bee (Figure 2). A droplet of lemon water was held to one side of the bee (order randomised) at the level of its eye for 5 sec, followed 30 sec later by a droplet of vanilla held on the same side of the bee for 5 sec. Each pair of odours was presented to the bee's left or right side. The left/right order was random. The bee's response was scored as in experiment 1. A third experiment repeated this procedure but controlled for visual cues by presenting a syringe with a droplet on both sides simultaneously, one droplet with lemon or vanilla odour and the other distilled water.
